# Her2/neu extracellular domain shedding in uterine serous carcinoma: implications for immunotherapy with trastuzumab

**DOI:** 10.1038/bjc.2011.369

**Published:** 2011-09-13

**Authors:** P Todeschini, E Cocco, S Bellone, J Varughese, K Lin, L Carrara, F Guzzo, N Buza, P Hui, D-A Silasi, E Ratner, M Azodi, P E Schwartz, T J Rutherford, S Pecorelli, A D Santin

**Affiliations:** 1 Department of Obstetrics, Gynecology and Reproductive Sciences, Yale University School of Medicine, Room 305 LSOG, PO Box 208063, 333 Cedar Street, New Haven, CT, USA; 2 Department of Pathology, Yale University School of Medicine, New Haven, CT, USA; 3 Nocivelli Institute for Molecular Medicine and Division of Gynecologic Oncology, University of Brescia, Brescia, Italy

**Keywords:** uterine serous cancer, Her2/neu, Her2/neu extracellular domain (ECD), trastuzumab

## Abstract

**Background:**

We evaluated shedding of epidermal growth factor type II receptor (Her2/neu) extracellular domain (ECD) in primary uterine serous carcinoma (USC) cell lines and in the serum of USC patients and its biological effects in experiments of trastuzumab-induced cytotoxicity *in vitro*.

**Methods:**

Her2/neu expression was evaluated by immunohistochemistry (IHC), real-time PCR and flow cytometry, while *c-erbB2* gene amplification was assessed using fluorescent *in situ* hybridisation (FISH). Her2/neu ECD levels in the supernatants of USC cell lines and in the serum of 38 USC patients and 19 controls were tested using ELISA. The biologic effect of Her2/neu ECD on trastuzumab-induced antibody-dependent cell-mediated cytotoxicity (ADCC) was evaluated in 5-h chromium-release assays.

**Results:**

Five out of ten USC cell lines overexpressed Her2/neu by IHC and showed amplification of the *c-erbB2* gene. High levels of Her2/neu ECD were found in supernatants of all FISH-positive tumours. In contrast, FISH-negative USC was negative for Her2/neu ECD shedding. Serum Her2/neu ECD levels in patients harbouring 3+Her2/neu tumours were higher than those found in healthy women (*P*=0.02) or USC patients with 2+ or 1+/negative Her2/neu expression (*P*=0.02). In cytotoxicity experiments, trastuzumab-mediated ADCC was significantly decreased by the addition of Her2/neu ECD-containing supernatants (*P*=0.01).

**Conclusion:**

FISH-positive c-erbB2 USC cell lines shed high levels of Her2/neu ECD. High levels of Her2/neu ECD in USC patients may reduce trastuzumab-mediated ADCC *in vitro* and potentially neutralise its therapeutic effect *in vivo*.

Endometrial cancer is the most common female genital tract malignancy in the United States, with an incidence of 43 470 new cases and 7950 deaths in 2010 (Jemal *et al*, 2010). Uterine serous carcinoma (USC) represents the most biologically aggressive subtype of endometrial cancer (Hendrickson *et al*, 1982). Although USC accounts for only 10% of all cases of endometrial carcinoma, it is responsible for ∼50% of all uterine cancer relapses and deaths (Hendrickson *et al*, 1982). Uterine serous carcinoma is characterised by a high propensity for early lymphovascular invasion, as well as intraperitoneal and extraabdominal spread at the time of presentation. The overall 5-year survival is about 30% for all stages and the recurrence rate after surgery is extremely high (50–80%) (Schwartz, 2006). Thus, there is a dire need for the development of novel, target specific and more effective therapeutic strategies against this rare subset of endometrial cancer.

*c-erbB2* is a proto-oncogene that encodes the human epidermal growth factor type II receptor (Her2/neu), a 185-kDa transmembrane protein, composed of three domains: the cytoplasmic tyrosine kinase domain responsible for intracellular signalling, a hydrophobic membrane-spanning region and the extracellular domain (ECD), which includes the binding site for trastuzumab (Slamon *et al*, 1989). Overexpression of Her2/neu has been found to be associated with resistance to chemotherapy and poor survival in multiple human malignancies, particularly in breast cancer, where Her2/neu has been most extensively studied (Wright *et al*, 1989; Lofts and Gullick, 1992).

Our group and others, including the Gynecologic Oncology Group in a cooperative multicentre study, have reported Her2/neu overexpression (i.e., 2+ and/or 3+ by immunohistochemistry, IHC) in 40–60% of patients harbouring USC (Santin *et al*, 2005a, 2005b, 2005c; Díaz-Montes *et al*, 2006; Morrison *et al*, 2006; Grushko *et al*, 2008). Furthermore, in previous reports, patients with USC tumours overexpressing Her2/neu receptor have been found to have a worse prognosis than those who do not (Santin *et al*, 2005a, 2005c; Morrison *et al*, 2006). These findings have provided a rationale to the use of Her2/neu-targeted therapies in patients harbouring this aggressive endometrial cancer subtype.

Trastuzumab (Herceptin, Genentech, San Francisco, CA, USA) is a humanised monoclonal antibody (mAb) directed against the ECD of Her2/neu. This therapeutic agent has been shown to be highly effective in patients with early or advanced/metastatic breast cancer overexpressing Her2/neu (Slamon *et al*, 2001; Baselga *et al*, 2005). Importantly, correlations have been reported between high baseline shedding of Her2/neu ECD and poor response to trastuzumab, suggesting that the ECD may represent a useful serum marker as a prognostic indicator and as a predictor of response to treatment in breast cancer patients (Hoopmann *et al*, 2003; Bethune-Volters *et al*, 2004). To our knowledge, however, no study has yet investigated whether biologically aggressive USC may shed Her2/neu ECD or whether Her2/neu ECD is detectable in the serum of USC patients. To fill this gap in knowledge, in this study we analysed 10 primary USC cell lines for Her2/neu receptor expression and *c-erbB2* gene amplification, and investigated Her2/neu ECD release in the supernatant of these biologically aggressive tumours. In addition, we have quantified the presence of soluble Her2/neu ECD in the serum of patients harbouring USC expressing different levels of Her2/neu. Finally, we have analysed the potential biologic effects of *in vivo* Her2/neu ECD shedding studying its effect in experiments of trastuzumab-induced cytotoxicity.

## Materials and methods

### Establishment of USC cell lines

Ten primary USC cell lines (USPC ARK-1 to USPC ARK-10) were established after sterile processing of tumour samples from surgical biopsy specimens, as described previously (El-Sahwi *et al*, 2010), under approval of the Institutional Review Board. Tumours were staged according to the International Federation of Gynecologists and Obstetricians 1988 operative staging system. Source-patient characteristics of these 10 USC cell lines are described in Table 1. BT-474 and SK-BR-3 human breast carcinoma control cell lines with Her2/neu overexpression and gene amplification were purchased from ATCC (Rockville, MD, USA).

### Her2/neu immunostaining of formalin-fixed tumour tissues

Formalin-fixed, paraffin-embedded tissue blocks from the USC patients from whom primary cell lines were established were retrieved from surgical pathology files. Specimens were reviewed by a gynecologic pathologist. The level of expression of Her2/neu was evaluated on the most representative block by standard immunohistochemical staining, using the Hercept test (Dako, Glostrup, Denmark), as previously described (El-Sahwi *et al*, 2010).

### Fluorescent *in situ* hybridisation of cell blocks obtained from primary USC

Fluorescent *in situ* hybridisation (FISH) analysis was performed in the tumour tissues using the PathVysion Her-2 DNA FISH Kit (Abbott Molecular Inc., Abbott Park, IL, USA) according to the manufacturer's instructions, as previously described (El-Sahwi *et al*, 2010).

### Quantitative real-time PCR

RNA isolation from all 10 primary USC cell lines and 2 breast cancer cell lines was performed using TRIzol Reagent (Invitrogen, Carlsbad, CA, USA), according to the manufacturer's instructions. Quantitative PCR was carried out with a 7500 RealTime PCR System using the manufacturer's recommended protocol (Applied Biosystems, Foster City, CA, USA) to evaluate the expression of *erbB2* in all samples. The primers and probe for *erbB2* were obtained from Applied Biosystems (Assay ID Hs00170433_m1). The comparative threshold cycle method was used to determine gene expression in each sample, relative to the value observed in the lowest non-malignant endometrial epithelial cell sample, using glyceraldehyde-3-phosphate dehydrogenase (Assay ID Hs99999905_m1) RNA as internal control.

### Flow cytometry

**Figure 1:**
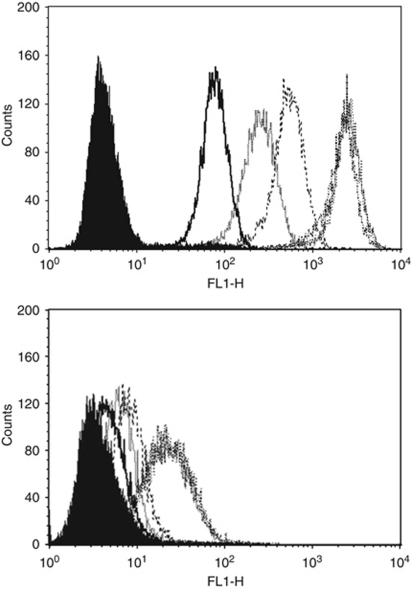
Representative flow cytometry histograms of trastuzumab dose titration (ranging from 0.05 to 1.5 *μ*g ml^−1^) in USPC ARK-2 and in USPC ARK-10. In multiple experiments, binding to USC cell lines (USPC ARK-2, upper panel; USPC ARK-10, lower panel) was found to plateau at a trastuzumab concentration between 0.5 and 1 *μ*g ml^−1^.

The clinically marketed anti-Her2/neu monoclonal antibody trastuzumab (Herceptin; Genentech) was used for our flow cytometry studies. For staining, a fluorescein isothiocyanate-conjugated goat antihuman F(ab1)_2_ immunoglobulin was used as a secondary reagent (BioSource International, Camarillo, CA, USA). Analysis was conducted with a FACScalibur, using Cell Quest software (BD Biosciences, San Diego, CA, USA). Using *in vitro* dose titration experiments with different amounts of trastuzumab (ranging from 0.05 to 1.5 *μ*g ml^−1^), binding of USC cells was found to plateau at a trastuzumab concentration ranging from 0.5 to 1 *μ*g ml^−1^ (Figure 1). On the basis of these results, we used 0.5 and 1 *μ*g ml^−1^ of trastuzumab in all of our cytotoxicity experiments.

### Analysis of Her2/neu ECD shedding in tumour samples

To evaluate the potential secretion of Her2/neu ECD by primary cancer cell lines, supernatants obtained from all 10 USC cell lines and 2 breast cancer cell lines were evaluated by a commercially available ELISA kit (eBioscience, Inc., San Diego, CA, USA). Briefly, tumour supernatants to be tested for Her2/neu ECD secretion were collected after incubation of 10^6^ primary tumour cells for each cell line cultured in plasticware (Corning, Corning, NY, USA) using RPMI-1640 media, supplemented with 10% FBS (Gemini, Woodland, CA, USA). After 96 h incubation at 37 °C, supernatants were aspirated, rendered cell free by centrifugation at 1500 r.p.m. for 10 min, and stored at −80 °C before being analysed for Her2/neu ECD by ELISA (see below). The Her2/neu ECD values are expressed in ng ml^−1^.

### Measurement of Her2/neu ECD concentration in serum samples

Her2/neu concentration was measured in the serum samples of 19 healthy female donors and 38 USC patients (i.e., 10 patients harbouring Her2/neu 3+ tumours, 14 patients with 2+ tumours and 14 patients with 1+ or negative tumours for Her2/neu expression by IHC), using an ELISA for the quantitative detection of human soluble Her2/neu protein, according to the manufacturer's instructions (Human sHER-2 eBioscience, Inc.). Serum was collected in all patients at the time of pre-operative visits before their surgery and before any treatment. Patient characteristics are described in Table 1.

### Preparation of conditioned media

Large-scale production of conditioned medium was obtained from supernatants of USPC ARK-2 cell line (i.e., a FISH-positive cell line). Cells were plated into T150 flasks in RPMI medium supplemented with 10% FBS for a total of 6 days. The medium was then collected and centrifuged at 1000 r.p.m. for 7 min to remove insoluble material. Conditioned medium was concentrated 40-fold by ultracentifugation using YM50 membranes (Millipore/Amicon, Billerica, MA, USA) before being used in cytotoxicity assays. Control supernatants collected from FISH-negative USC cell lines were similarly processed before their use.

### Antibody-dependent cell-mediated cytotoxicity measurement

A standard 5-h chromium (^51^Cr)-release assay was performed to measure the cytotoxic reactivity of Ficoll-Paque PLUS (GE Healthcare, Uppsala, Sweden) separated peripheral blood lymphocytes (PBLs) obtained from several healthy donors against two representative FISH-positive primary USC cell lines (i.e., USPC ARK-2 and USPC ARK-10), as previously described (El-Sahwi *et al*, 2010). To analyse the biological effect of soluble Her2/neu ECD on trastuzumab-mediated antibody-dependent cell-mediated cytotoxicity (ADCC), we incubated trastuzumab (final concentrations 0.5 or 1 *μ*g ml^−1^) with conditioned medium containing 500 ng ml^−1^ of soluble Her2/neu ECD or matched control supernatants negative for soluble Her2/neu ECD for 1 h at RT on a rocker. The antibody/supernatant preparations were then added to the target cells (USPC ARK-2 and USPC ARK-10) for 30 min before incubation with the effector PBLs. Controls included the incubation of target cells alone or with PBL or mAb separately. The chimeric anti-CD20 mAb rituximab was used as an additional negative control in the assays.

### Statistical analysis

For qRT–PCR data, the right skewing was removed by taking copy number ratios relative to the lowest expressing normal human endometrial cells sample (‘relative copy number’), log_2_ transforming them to *ΔC*_t_s, and comparing the results through unequal-variance *t*-test for FISH-positive *vs* FISH-negative USC. Group means with 95% confidence intervals (CIs) were calculated by computing them on the *ΔC*_t_s and then reverse transforming the results to obtain means (95% CIs) of relative copy numbers. The analyses of differences in Her2/neu ECD shedding in supernatants and in Her2/neu expression levels measured by flow cytometry and IHC were performed using the Wilcoxon–Mann–Whitney test. Kruskal–Wallis test and *χ*^2^-analysis were used to evaluate differences in trastuzumab-mediated ADCC levels in primary tumour cell lines. Her2/neu serum concentrations among the different groups of patients (i.e., healthy controls, USC 3+, USC 2+ and USC 1+/negative) were summarised as means and compared with Student's *t*-test. Statistical analysis was performed using PASW version 18 (SPSS, Chicago, IL, USA). A *P*-value of <0.05 was considered statistically significant.

## Results

### Her2/neu expression by IHC on USC

Immunohistochemistry detecting Her2/neu expression was performed on formalin-fixed paraffin-blocks of USC tissues from which the 10 primary cell lines were established. As reported in Table 2, 5 out of 10 specimens showed strong staining (3+) for Her2/neu protein, whereas the remaining five showed weak-to-moderate staining (1+ and 2+).

### Fluorescent *in situ* hybridisation

FISH analysis was performed on the cell blocks obtained from USPC ARK-3 and USPC ARK-6 cell lines and on formalin-fixed paraffin-embedded tissue blocks from the other eight USCs used in this study. c-*erbB2* gene amplification was detected in 5 out of 10 primary USC specimens, suggesting that strong receptor expression by IHC and high Her2/neu mRNA level of these tumours (see below) are likely caused by gene amplification. In contrast, the remaining five USC cell lines were found to be negative for c-*erbB2* gene amplification (Table 2).

### qRT–PCR

A total of 10 primary USC cell lines and 2 breast cancer cell lines (BT-474 and SK-BR-3) were tested by real-time PCR for evaluating the expression of Her2/neu at mRNA level. High levels of Her2/neu mRNA transcripts were detected in five out of five (100%) of the FISH-positive cell lines tested, with values ranging from 549.8 to 4993.8 (Table 2). In contrast, low-to-moderate Her2/neu expression was detected in the other five FISH-negative cell lines, with values ranging from 17.6 to 95.3 (Table 2). These data are in full agreement with the results obtained by IHC. Breast cancer cell lines BT-474 and SK-BR-3 were also found to express high level of Her2/neu mRNA copy numbers (i.e., 897 (BT-474) and 932 (SK-BR-3); Table 2).

### Flow cytometry

Surface Her2/neu expression was evaluated by FACS analysis on all 10 primary USC cell lines and 2 breast cancer cell lines (BT-474 and SK-BR-3) using trastuzumab. In addition, as negative controls, several B-cell lines (EBV-transformed lymphoblastoid B-cell lines) established from the same USC patients from which the tumour cell lines had been established were also studied (data not shown). Four out of ten USC cell lines (all FISH positive) showed a very high expression of Her2/neu (mean fluorescence intensity (MFI) ranging from 228 to 710), while 6 out of 10 (1 FISH positive and 5 FISH negative) were found to express significantly lower levels of Her2/neu (MFI ranging from 10 to 47) (Table 2, *P*>0.01). USPC ARK-10, a FISH-positive tumour, was particularly remarkable because the gene amplification and high transcript expression levels (FISH ratio 4.3, mRNA copy number=549.8) did not correlate with a strong surface receptor expression by flow cytometry. Both breast cancer cell lines were found to express high levels of Her2/neu protein by flow cytometry with an MFI of 654 (BT-474) and 265 (SK-BR-3) (Table 2).

### Her2/neu ECD levels in supernatants of primary USC and breast cancer cell lines

Cell-free supernatants obtained from all 10 USC cell lines and 2 breast cancer cell lines were collected and analysed for quantitative detection of soluble Her2/neu ECD by ELISA. We found high secretion of Her2/neu ECD (mean 4.6 × 10^−6^ ng ml^−1^, range 1.6–9.1 × 10^−6^ ng ml^−1^ per 96 h) in five out of five of the FISH-positive primary USC tested. In contrast, Her2/neu ECD was undetectable in the supernatants of the five Her2/neu low-expressing cell lines. SK-BR-3 and BT-474 tumours were found to secrete large amounts of Her2/neu ECD, 6.4 × 10^−6^ ng ml^−1^ and 2.9 × 10^−6^ ng ml^−1^ per 96 h, respectively.

### Serum Her2/neu ECD levels in USC patients and healthy donors

Serum from healthy female donors and USC patients harbouring tumours with different Her2/neu expression was tested for Her2/neu ECD levels by ELISA. No significant differences were found in the serum levels of Her2/neu ECD between healthy donors (mean±s.d.: 6.3±3.36, range 2–12.26 ng ml^−1^) and patients harbouring Her2/neu 2+ USC (mean±s.d.: 6.61±2.37, range 3.44–12.20 ng ml^−1^) or patients harbouring Her2/neu 1+/negative USC (mean±s.d.: 6.27±2.29, range 4.21–12.42 ng ml^−1^). In contrast, the serum Her2/neu ECD levels of the 10 patients harbouring Her2/neu 3+ tumours had a mean±s.d. of 13.18±7.81, ranging from 6.56 to 28.80 ng ml^−1^ (Table 3); these values were statistically significantly higher than those of the healthy group (*P*=0.02), or the group of Her2/neu 2+ or 1+/negative USC patients (*P*=0.02).

### Antibody-dependent cell-mediated cytotoxicity

**Figure 2:**
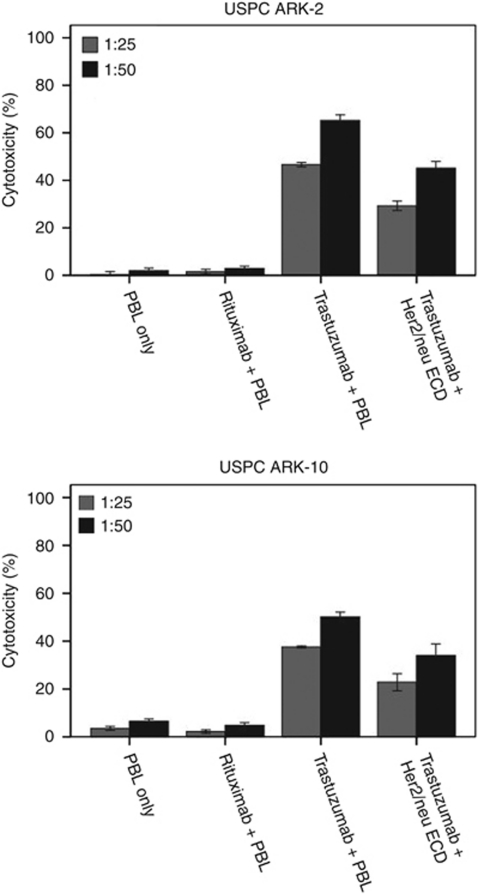
Representative cytotoxicity experiments using trastuzumab alone and trastuzumab preincubated with Her2/neu ECD (500 ng ml^−1^) against Her2/neu-positive USC cell lines (USPC ARK-2 and USPC ARK-10) (E/T ratio 50:1 and 25:1). Trastuzumab-mediated ADCC was significantly decreased after incubation with Her2/neu ECD in both cell lines (*P*=0.01).

USPC ARK-2 and USPC ARK-10, two Her2/neu FISH-positive tumours shedding Her2/neu ECD in the supernatants but showing different levels of Her2/neu surface expression by flow cytometry (Table 2), were representatively tested for their sensitivity to natural killer (NK) cytotoxicity when challenged with heterologous PBLs collected from several healthy donors in a standard 5-h ^51^Cr-release assay. Both cell lines were found to be resistant to NK-mediated cytotoxicity when combined with PBLs at effector to target cell ratios (E:T) varying from 25:1 and 50:1 (range of cytotoxicity from 0% to 8.4% with all E:T, Figure 2). Similarly, both USC cell lines incubated with rituximab control antibody and PBL were not significantly killed (range from 0% to 8.5%, Figure 2). In contrast, both USC cell lines were found to be highly sensitive to trastuzumab-mediated ADCC (ranges of killing for USPC ARK-2=46–69% mean killing±s.d.=46.6±1.6 at 25:1 ratio and 65.4±4.0 at 50:1 ratio, and USPC ARK-10, ranges of killing of 37–54%, mean killing±s.d.=37.6±0.7 at 25:1 ratio and 50.2±3.3 at 50:1 ratio, respectively). We then evaluated the sensitivity of USPC ARK-2 and USPC ARK-10 cell lines to NK cytotoxicity and trastuzumab in the presence or absence of the Her2/neu ECD. Interaction of trastuzumab with supernatant containing Her2/neu ECD before incubation with the effector PBLs, significantly reduced NK-mediated killing in both the cell lines (ranges of killing for USPC ARK-2=25–50% mean killing±s.d.=29.2±3.5 at 25:1 ratio and 45.1±4.9 at 50:1 ratio, and USPC ARK-10, ranges of killing of 18–41.8%, mean killing±s.d.=22.8±6.2 at 25:1 ratio and 34.0±8.3 at 50:1 ratio, respectively, *P*=0.01, Figure 2). In contrast, incubation of trastuzumab with controls supernatants (collected from FISH-negative USC cell lines) had no effect on ADCC (data not shown).

## Discussion

Our research group has recently examined Her2/neu expression, gene amplification, and its association with outcome in USC, the most aggressive and chemotherapy-resistant variant of endometrial carcinoma (Santin *et al*, 2002, 2005a, 2005b, 2005c, 2008; El-Sahwi *et al*, 2010). Her2/neu overexpression and *c-erbB2* gene amplification were detected in a significant number of USC patients and found to be associated with a more aggressive biological behaviour and poorer prognosis (Santin *et al*, 2005a, 2005c; Morrison *et al*, 2006; Grushko *et al*, 2008). Importantly, these findings have led to consideration of Her2/neu as a potential marker for trastuzumab-based therapy (Santin *et al*, 2002, 2008; Villella *et al*, 2006). As reported in breast cancer, where trastuzumab has been demonstrated to have significant therapeutic effect in patients with strong (i.e., score 3+ by IHC) or FISH-positive disease (Slamon *et al*, 2001, 2006; Baselga *et al*, 2005), USC patients refractory to standard treatment modalities may potentially benefit from Her2/neu-targeted therapy (Villella *et al*, 2006; Santin *et al*, 2008). Consistent with this hypothesis, a prospective randomised clinical trial comparing the efficacy of trastuzumab in combination with carboplatin and paclitaxel *vs* carboplatin and paclitaxel alone in advanced/recurrent USC patients overexpressing Her2/neu is currently recruiting patients at Yale University.

In the current study, using multiple techniques we demonstrated high Her2/neu expression in 5 out of 10 primary USC cell lines established in our laboratory. We also confirmed that USC cell lines overexpressing Her2/neu are sensitive to NK-mediated cytotoxicity in the presence of trastuzumab (Santin *et al*, 2002; El-Sahwi *et al*, 2010). Importantly, because of the lack of previous information regarding potential Her2/neu ECD shedding by primary USC cell lines or presence of Her2/neu ECD in the serum samples of patients harbouring USC, we investigated Her2/neu ECD release in the supernatants of multiple primary USC cell lines and in the serum of USC patients and studied Her2/neu ECD's potential biological effects in experiments of trastuzumab-induced cytotoxicity *in vitro*. We report, to our knowledge for the first time, high levels of Her2/neu ECD by ELISA in the supernatants of five out of five (100%) USC cell lines overexpressing Her2/neu at 3+ levels and harbouring *c-erbB2* gene amplification. In contrast, no Her2/neu ECD was detectable in the supernatants of the remaining five USPC cell lines that did not overexpress Her2/neu. Consistent with these *in vitro* data obtained using primary cell lines, patients harbouring USC overexpressing Her2/neu at 3+ levels were found to have significantly higher levels of Her2/neu ECD in their serum samples when compared with healthy women or patients harbouring tumours with moderate (i.e., 2+) or low/negative (i.e., 1+/negative) Her2/neu expression by IHC (*P*=0.02). In contrast, no difference was found in serum Her2/neu ECD levels between healthy donors and patients harbouring USC with moderate or low/negative Her2/neu expression.

Alper *et al* (1990) were the first to report the presence of Her2/neu ECD in the culture medium conditioned by the breast cancer cell line SK-BR-3. Subsequently, the ECD of the Her2/neu protein was shown to be proteolytically cleaved by metalloproteases from the surface of the cells and released in the supernatants of the culture media *in vitro* and also detectable in the circulation of patients with breast cancer (Zabrecky *et al*, 1991; Pupa *et al*, 1993; Wu *et al*, 1995). Within the context of trastuzumab-based therapy, the measurement of serum Her2/neu ECD has generated an increasing interest to understand the precise biological function of the soluble Her2/neu portion in cancer development as well as its clinical implications in the management of patients eligible for trastuzumab therapy, both as a prognostic marker and as a predictor of response to treatment.

The relationship between baseline serum Her2/neu ECD concentration and benefit from trastuzumab-based treatment in breast cancer patients, however, is currently controversial. Several studies have found no predictive value of pretreatment serum Her2/neu ECD levels both in primary and in metastatic breast cancer (reviewed by Leary *et al*, 2009). In contrast, others have shown that elevated pretreatment serum levels of Her2/neu ECD may be predictive for improved response to trastuzumab-based therapy (Kostler *et al*, 2004). Ghedini *et al* (2010) has recently shown in an animal model, that increased circulating levels of recombinant Her2/neu ECD (rECD) may indeed decrease and delay Her2-positive tumour growth in a Her2/neu genetically engineered ovarian cancer model. Furthermore, they demonstrated that rECD could increase the binding site for trastuzumab through heterodimerisation with other receptors of the HER family (Ghedini *et al*, 2010). Conflicting with these results, however, other groups have reported that low baseline Her2/neu serum levels predict greater benefit from trastuzumab-based treatment (Hoopmann *et al*, 2003; Bethune-Volters *et al*, 2004) and that elevated pretreatment Her2/neu ECD levels may predict poor response to both hormone therapy and adjuvant chemotherapy (Colomer *et al*, 2000; Lipton *et al*, 2002).

In this context, the truncated form of the Her2/neu receptor, known as p95Her2/neu, that arises through the proteolytic shedding of the ECD of the full-length Her2/neu by alternative initiation of translation and alternative RNA processing, has been previously identified as a potential mechanism to explain resistance to treatment of Her2/neu ECD shedding tumours. Consistent with this view, the remaining membrane-bound portion (p95) of Her2/neu has a constitutively activated kinase domain and its presence has been correlated with poor prognosis and decreased responsiveness to treatments (Scott *et al*, 1993; Anido *et al*, 2006). Based on these controversial conclusions, the clinical usefulness of Her2/neu ECD is still largely unknown in breast cancer and further studies are needed to clarify the real clinical implications of Her2/neu ECD shedding in the context of trastuzumab-based therapy.

Although multiple mechanisms of action have been attributed to trastuzumab including prevention of Her2-receptor dimerisation, inhibition of tumour proliferation, increased endocytotic destruction of the receptor and inhibition of shedding of the ECD, strong experimental evidence suggests that engagement of Fc receptors on effector cells (i.e., mainly NK cells) represents the dominant component of the *in vivo* activity of trastuzumab (Clynes *et al*, 2000). Consistent with this view, in preclinical tumour models of anti-Her2/neu-based mAb therapies, mice deficient in activating Fc receptors as well as antibodies engineered to disrupt Fc binding to those receptors were unable to arrest tumour growth *in vivo* (Clynes *et al*, 2000). Accordingly, in an attempt to understand the possible implication of high levels of Her2/neu ECD shedding during trastuzumab-induced cytotoxicity in USC patients, in our study we have compared trastuzumab ADCC in the absence or presence of the soluble portion of Her2/neu against primary Her2/neu FISH-positive USC cell lines showing different levels of surface Her2/neu expression. We found a consistent and significant decrease in trastuzumab-mediated tumour cell killing against both FISH-positive cell lines used in our experiments (*P*=0.01). Our results are, therefore, in agreement with those published by Brodowicz *et al* (1997) using established breast cancer cell lines, showing that the soluble form of the extracellular Her2/neu could block lymphokine-activated killer cell-dependent cytolysis of the same tumour cells. Of interest, we found the decrease in trastuzumab-mediated killing of the USPC ARK-10 cell line to be more dramatic when compared with the USPC ARK-2 cell line. These results are likely explained by a lower expression of the Her2/neu protein on the surface of the USPC ARK-10 when compared with USPC ARK-2, regardless of the fact that both primary tumours were FISH positive and able to shed Her2/neu EDC. Taken together, these data suggest that high amount of soluble Her2/neu ECD may significantly reduce the activity of trastuzumab by antibody sequestration and subsequently decrease NK-mediated killing of USC. We are tempted to speculate that these findings may help to explain why some patients with FISH-positive tumours who shed Her2/neu EDC but express relatively low levels of surface Her2/neu may experience suboptimal responses to trastuzumab therapy *in vivo*.

In conclusion, our results show for the first time that primary USC cell lines that overexpress Her2/neu release high amounts of Her2/neu ECD in the culture media and soluble ECD may significantly block one of the most effective mechanisms of trastuzumab activity (i.e., trastuzumab-mediated cytotoxicity) *in vitro*. Furthermore, we demonstrated that patients harbouring high Her2/neu-expressing USC have high amounts of Her2/neu ECD in the circulation. On the basis of these results, prospective evaluation of the biological function of Her2/neu ECD in USC patients overexpressing Her2/neu undergoing trastuzumab-based therapy seems warranted.

## Figures and Tables

**Table 1:** Patients characteristics from which the 10 cell lines were established

**Patient**	**Age (years)**	**Race**	**Stage**	**Histopathology**
USPC ARK-1	62	AA	IVA	Pure
USPC ARK-2	63	AA	IVB	Pure
USPC ARK-3	59	AA	IVB	Mixed
USPC ARK-4	73	C	IVB	Pure
USPC ARK-5	73	AA	IIIC	Pure
USPC ARK-6	62	C	IB	Mixed
USPC ARK-7	75	C	IIC	Pure
USPC ARK-8	88	C	IIIA	Pure
USPC ARK-9	73	AA	IIIC	Mixed
USPC ARK-10	79	C	IVB	Pure

Abbreviations: AA=African American; C=Caucasian; USPC=uterine serous papillary adenocarcinoma.

**Table 2:** Her2/neu expression and Her2/neu ECD release in the media supernatants of uterine serous tumours

			**RT–PCR**	**Flow cytometry**		**sHer2/neu**
	**IHC**	**FISH**	**mRNA copy no.**	**% Gated**	**MFI**	**ng ml^−1^ per 96 h**
USPC-ARK-1	3+	2.5^a^	2749.5	99.8	339.5	4.2 × 10^−6^
USPC-ARK-2	3+	5.2^a^	4478.9	99.9	710.3	4.9 × 10^−6^
USPC-ARK-3	3+	4.7^a^	4993.8	99.9	228.7	3.4 × 10^−6^
USPC-ARK-9	3+	4.6^b^	4326.5	99.3	285.7	9.1 × 10^−6^
USPC-ARK-10	3+	4.3^b^	549.8	98.8	25.4	1.6 × 10^−6^
USPC-ARK-7	2+	1.3^b^	45.6	94.9	47.4	0
USPC-ARK-6	1+	0.9^a^	44.3	93.4	13.0	0
USPC-ARK-8	1+	1.6^b^	17.6	83.2	24.8	0
USPC-ARK-4	1+	1.6^a^	52.8	95.2	10.8	0
USPC-ARK-5	1+	1.4^a^	95.3	83.8	18.7	0
BT-474	3+	Amplified	897	99.9	654.9	6.4 × 10^−6^
SK-BR-3	3+	Amplified	932	99.9	265.4	2.89 × 10^−6^

Abbreviations: FISH=fluorescent *in situ* hybridisation; IHC=immunohistochemistry; RT–PCR=real-time PCR; MFI=mean fluorescence intensity; USPC=uterine serous papillary adenocarcinoma; sHer2/neu=soluble Her2/neu.

a FISH analysis performed on formalin-fixed, paraffin-embedded cell blocks obtained from primary cell lines in culture.

b FISH performed on formalin-fixed, paraffin-embedded tissue blocks of the original tumour sample.

**Table 3:** Circulating levels of Her2/neu ECD (ng ml^−1^) in healthy donors and USC patients harbouring tumours with different levels of Her2/neu expression

**3+**	**2+**	**1+/neg**	**Healthy donors**
7.84	3.97	9.10	6.34
8.20	3.44	4.72	12.16
6.56	5.76	4.32	12.26
12.26	10.00	5.30	4.96
21.54	5.25	7.44	6.18
9.00	12.20	6.53	9.68
8.69	5.46	12.42	8.08
21.38	4.80	4.60	11.12
28.80	4.93	4.74	6.42
7.50	7.25	4.21	4.4
	6.56	6.10	6.02
	7.54	6.00	10.28
	7.6	4.24	4.08
	7.73	6.80	2.98
			3.62
			3.18
			3.79
			2.17
			2
			
*Mean±s.d.*			
13.18±7.81	6.61±2.37	6.27±2.29	6.3±3.36
			
*Patient number*			
10	14	14	19

Abbreviations: ECD=extracellular domain; USC=uterine serous carcinoma.
